# Comparative genomic analysis of NAC transcriptional factors to dissect the regulatory mechanisms for cell wall biosynthesis

**DOI:** 10.1186/1471-2105-13-S15-S10

**Published:** 2012-09-11

**Authors:** Dongxia Yao, Qiang Wei, Wenying Xu, Ryan D Syrenne, Joshua S Yuan, Zhen Su

**Affiliations:** 1 State Key Laboratory of Plant Physiology and Biochemistry, College of Biological Sciences, China Agricultural University, Beijing 100193, China; 2 Department of Plant Pathology and Microbiology, Texas A&M University, College Station, TX 77843, USA

## Abstract

**Background:**

NAC domain transcription factors are important transcriptional regulators involved in plant growth, development and stress responses. Recent studies have revealed several classes of NAC transcriptional factors crucial for controlling secondary cell wall biosynthesis. These transcriptional factors mainly include three classes, SND, NST and VND. Despite progress, most current analysis is carried out in the model plant *Arabidopsis*. Moreover, many downstream genes regulated by these transcriptional factors are still not clear.

**Methods:**

In order to identify the key homologue genes across species and discover the network controlling cell wall biosynthesis, we carried out comparative genome analysis of NST, VND and SND genes across 19 higher plant species along with computational modelling of genes regulated or co-regulated with these transcriptional factors.

**Results:**

The comparative genome analysis revealed that evolutionarily the secondary-wall-associated NAC domain transcription factors first appeared in *Selaginella moellendorffii*. In fact, among the three groups, only VND genes appeared in *S. moellendorffii*, which is evolutionarily earlier than the other two groups. The *Arabidopsis *and rice gene expression analysis showed specific patterns of the secondary cell wall-associated NAC genes (SND, NST and VND). Most of them were preferentially expressed in the stem, especially the second internodes. Furthermore, comprehensive co-regulatory network analysis revealed that the SND and MYB genes were co-regulated, which indicated the coordinative function of these transcriptional factors in modulating cell wall biosynthesis. In addition, the co-regulatory network analysis revealed many novel genes and pathways that could be involved in cell wall biosynthesis and its regulation. The gene ontology analysis also indicated that processes like carbohydrate synthesis, transport and stress response, are coordinately regulated toward cell wall biosynthesis.

**Conclusions:**

Overall, we provided a new insight into the evolution and the gene regulatory network of a subgroup of the NAC gene family controlling cell wall composition through bioinformatics data mining and bench validation. Our work might benefit to elucidate the possible molecular mechanism underlying the regulation network of secondary cell wall biosynthesis.

## Background

As a potential replacement for traditional fossil fuels, biofuels have received increased public and scientific attention in recent years [1]. The current first generation biofuel is based on sugar and starch derived from feedstocks such as sugrarcane and corn; however, this platform is not sustainable for various reasons. Lignocellulosic biomass has been proposed as the major feedstock for second generation biofuels to enable the transition from fossil fuel-based energy to renewable energy for the various economic and environmental advantages gained over first generation biofuels [1,2]. Generally speaking, lignocellulosic biomass is composed of cellulose, hemicellulose, pectin and/or lignin, but the amount and ratios between the components can vary considerably [3]. In addition to the aforementioned components, even the amorphous portions of cellulose are purportedly important for lignocellulosic conversion to biofuel [4-7]. Since secondary cell walls in fibres and tracheary elements constitute the most abundant biomass produced by plants, it is necessary to elucidate the possible molecular mechanisms underlying the regulation of secondary cell wall biosynthesis for improved plant biomass production.

Plant NAC (NAM, ATAF1/2 and CUC2) domain proteins are one of the largest groups of plant-specific transcriptional factors and are known to play diverse roles in various plant development processes and stress response. NAM (no apical meristem) was the first characterized NAC gene in petunia. The NAM gene product is required for apical meristem formation and correct positioning of the cotyledons during petunia embryogenesis [8]. ATAF1 and ATAF2 are the two NAC genes in *Arabidopsis *playing negative roles in response to drought and pathogen infection respectively [9,10]. CUC2 (CUP-SHAPED COTYLEDON 2) gene was also characterized as a NAC gene in *Arabidopsis *[11]. *Arabidopsis *RD26 (RESPONSIVE TO DEHYDRATION 26) encodes a NAC domain protein [12] with function in ABA-mediating gene expression under stress conditions [13]. StNAC, one potato NAC gene, was shown to be rapidly and strongly induced by wounding [14]. Over-expression of OsNAC6/SNAC2 in rice can enhance the seedling plants tolerance to drought, salt, and cold stresses [15,16]. Recently, accumulating evidence has indicated that a considerable portion of NAC domain proteins play crucial roles in the processes of xylogenesis, fibre development and wood formation in vascular plants [17]. In the model plant *Arabidopsis*, NST1 (NAC Secondary Wall Thickening Promoting Factor1), NST2 and NST3/SND1 (Secondary Wall-associated NAC Domain Protein1) are key switches in regulating secondary cell wall biosynthesis in a partially redundant manner [18-25]. NST1 and NST2 function redundantly in regulating secondary cell wall thickening in the endothecium of anthers whereas NST1 and NST3/SND1 were shown to regulate secondary wall thickening in fibres. In *Medicago sativa*, MtNST1 (Medicago truncatula NAC Secondary Wall Thickening Promoting Factor 1) has been identified as the only homologue of AtNSTs [26]. Loss of function of the single *MtNST1 *gene resulted in lack of lignifications in interfascicular fibres, loss of anther dehiscence and stomatal phenotypes associated with loss of ferulic acid in guard cell walls. VND6 (Vascular-related NAC-domain6) and VND7 are key regulators in protoxylem and metaxylem development. VND6 is specifically expressed in the metaxylem of *Arabidopsis *primary roots whereas VND7 is expressed in the protoxylem. Recently, the *VND6 *gene was discovered to regulate xylem formation by directly targeting some genes related to secondary cell wall formation. VND6 also acts as a direct regulator of genes related to programmed cell death [27]. SND2 and SND3 were also found to function in the formation of secondary cell walls in fibres, and were down-stream of NST1 and NST3/VND1. Six NAC genes associated with wood formation in *Populus *were also reported [28]. Among the six genes, WND2B (Wood-Associated NAC Domain Transcription Factors) and WND6B were functional orthologues of *Arabidopsis *SND1 and master switches activating secondary wall biosynthesis during wood formation in *Populus*. Recently, XND1(Xylem NAC Domain1) was reported to influence the differentiation of tracheary elements and xylem development in *Arabidopsis *by negatively regulating terminal secondary wall biosynthesis and programmed cell death in xylem vessel cells [29].

Although several key switches in regulation of secondary wall formation have been found in the model plants *Arabidopsis *and *Populus*, key regulators in other plants and many downstream genes regulated by these transcriptional factors are still not clear. In order to identify key homologue genes and discover the network controlling cell wall biosynthesis, we carried out comparative genome analysis of NST, VND and SND genes across 19 higher plant species. The analysis revealed that the NAC domain transcription factors associated with the secondary cell wall evolutionarily first appeared in *Selaginella moellendorffii*. In fact, among the three groups, only VND genes were identified in *S. moellendorffii*, which is evolutionarily earlier than the other two groups. Gene expression analysis was carried out to analyse the regulation of NAC genes associated with secondary cell wall biosynthesis in different tissues and revealed that several of these transcriptional factors were co-regulated. To further characterize the candidate genes involved in the regulation of secondary cell wall biosynthesis, we performed a comprehensive co-regulatory network analysis and discovered that some secondary wall-associated NAC genes and MYB genes were co-regulated. In addition, co-regulatory network analysis also revealed many novel genes and pathways that may be involved in cell wall biosynthesis and regulation.

## Methods

### Sequence retrieval and phylogenetic analysis

Protein sequences and DNA binding domain alignment of the NAC transcriptional factor gene family were downloaded from Plant Transcriptional Factor Database http://planttfdb.cbi.pku.edu.cn. Multiple alignments were performed using ClustalX (1.83) software, and the Neighbour-Joining (NJ) method was used to construct a phylogenetic tree. Genes sharing the same clade with the NAC genes controlling cell wall composition from *Arabidopsis *were chosen for further study, which resulted in 199 proteins across 19 species.

### Microarray data analysis

The expression profiling data was acquired from local and publically available databases (e.g. GEO and AtGenExpress). The signal intensity for each probe set of each GeneChip was extracted by Affymetrix software GCOS (MAS 5.0).

Eisen's cluster software http://rana.lbl.gov/EisenSoftware.htm was applied for cluster analysis. The signal intensities of microarray experiments were directly used for hierarchical clustering analysis. We employed standard data adjustment and SOM (Self-Organizing Map) clustering in precedence of hierarchical clustering to achieve a better grouping result.

Gene ontology (GO) analysis was performed for differentially expressed genes using the EasyGO web server [30]. During GO processing, the statistical test method used was the chi-square test with FDR p-value ≤ 0.05 as the cut-off.

The gene network data was constructed using Pathway Studio http://www.ariadnegenomics.com/products/pathway-studio/, ATTED http://atted.jp/ and Hans J. Bohnert's paper [31], and the map was constructed using Pathway Studio (version 6.2).

### Plant materials

Seven tissue samples of rice (*Oryza sativa *subsp. *japonica *var. Nipponbare) were selected for real-time RT-PCR (reverse transcription polymerase chain reaction) analysis. Rice calli were cultured in N6 solid medium [32] and harvested after one month of induction. Root samples were harvested from rice seedlings that were cultured in a growth container for two weeks. The other five samples (penultimate leaf, flag leaf, spikelet, seed and stem) were harvested from rice plant grown for about four months under natural conditions in Beijing, China.

### RNA isolation and real-time RT-PCR

All rice tissue samples were homogenized in liquid nitrogen before isolation of RNA. Total RNA was isolated using TRIZOL reagent (Invitrogen, CA, USA) and purified using Qiagen RNeasy columns (Qiagen, Hilden, Germany). Reverse transcription was performed using Moloney murine leukemia virus (M-MLV; Invitrogen). The cDNA samples were diluted to 8 ng/μL for real-time RT-PCR analysis.

For real-time RT-PCR, triplicate assays were performed on 1 μL of each cDNA dilution using the SYBR Green Master Mix (Applied Biosystems, USA, PN 4309155) with an ABI 7900 sequence detection system according to the manufacture's protocol (Applied Biosystems). The gene-specific primers were designed by using PRIMER3 http://frodo.wi.mit.edu/primer3/input.htm. The amplification of 18S rRNA was used as an internal control to normalize all data (forward primer, 5'-CGGCTACCACATCCAAGGAA-3' and reverse primer, 5'- TGTCACTACCTCCCCGTGTCA-3'). Gene-specific primers are listed in Supplemental Table 1 in additional file 1. The relative quantification method (ΔΔCT) [33] was used to evaluate quantitative variation between replicates examined.

**Table 1:** Gene Ontology enrichment analysis for the gene list in SNDs-related network

GO acc num	GO type	GO term	Query item	Bg item	FDR
GO:0009834	P	secondary cell wall biogenesis	12	18	2.40E-22
GO:0009832	P	plant-type cell wall biogenesis	13	52	8.00E-18
GO:0045449	P	regulation of transcription	17	231	1.70E-14
GO:0007275	P	multicellular organismal development	18	507	3.40E-10
GO:0048856	P	anatomical structure development	13	330	3.50E-08
GO:0044036	P	cell wall macromolecule metabolic process	6	40	1.90E-07
GO:0033692	P	cellular polysaccharide biosynthetic process	7	77	4.30E-07
GO:0009699	P	phenylpropanoid biosynthetic process	5	33	2.50E-06
GO:0010033	P	response to organic substance	9	210	3.40E-06
GO:0006355	P	regulation of transcription, DNA-dependent	14	770	7.20E-05
GO:0006950	P	response to stress	8	456	5.60E-03
GO:0048513	P	organ development	9	639	1.20E-02
GO:0003006	P	reproductive developmental process	9	740	3.10E-02
GO:0006810	P	transport	7	540	5.00E-02
GO:0003700	F	transcription factor activity	27	46	3.00E-49
GO:0016563	F	transcription activator activity	14	24	1.10E-25
GO:0008471	F	laccase activity	6	17	3.00E-09
GO:0016682	F	oxidoreductase activity, acting on diphenols and related substances as donors, oxygen as acceptor	6	28	6.70E-08
GO:0016679	F	oxidoreductase activity, acting on diphenols and related substances as donors	6	37	2.70E-07
GO:0046914	F	transition metal ion binding	8	195	3.50E-05
GO:0016757	F	transferase activity, transferring glycosyl groups	11	465	8.90E-05
GO:0016491	F	oxidoreductase activity	11	555	4.00E-04
GO:0043169	F	cation binding	8	306	5.30E-04
GO:0004553	F	hydrolase activity, hydrolyzing O-glycosyl compounds	8	397	2.90E-03
GO:0009055	F	electron carrier activity	5	264	4.10E-02
GO:0005634	C	nucleus	21	212	6.00E-20
GO:0044425	C	membrane part	8	364	3.30E-03
**Total**			**131**	**31819**	

P: biological process; F: molecular function; C: cellular component; Bg: background

## Results and discussion

### Identification of genes of NAC transcriptional factors controlling the cell wall composition across different species

**Figure 1:**
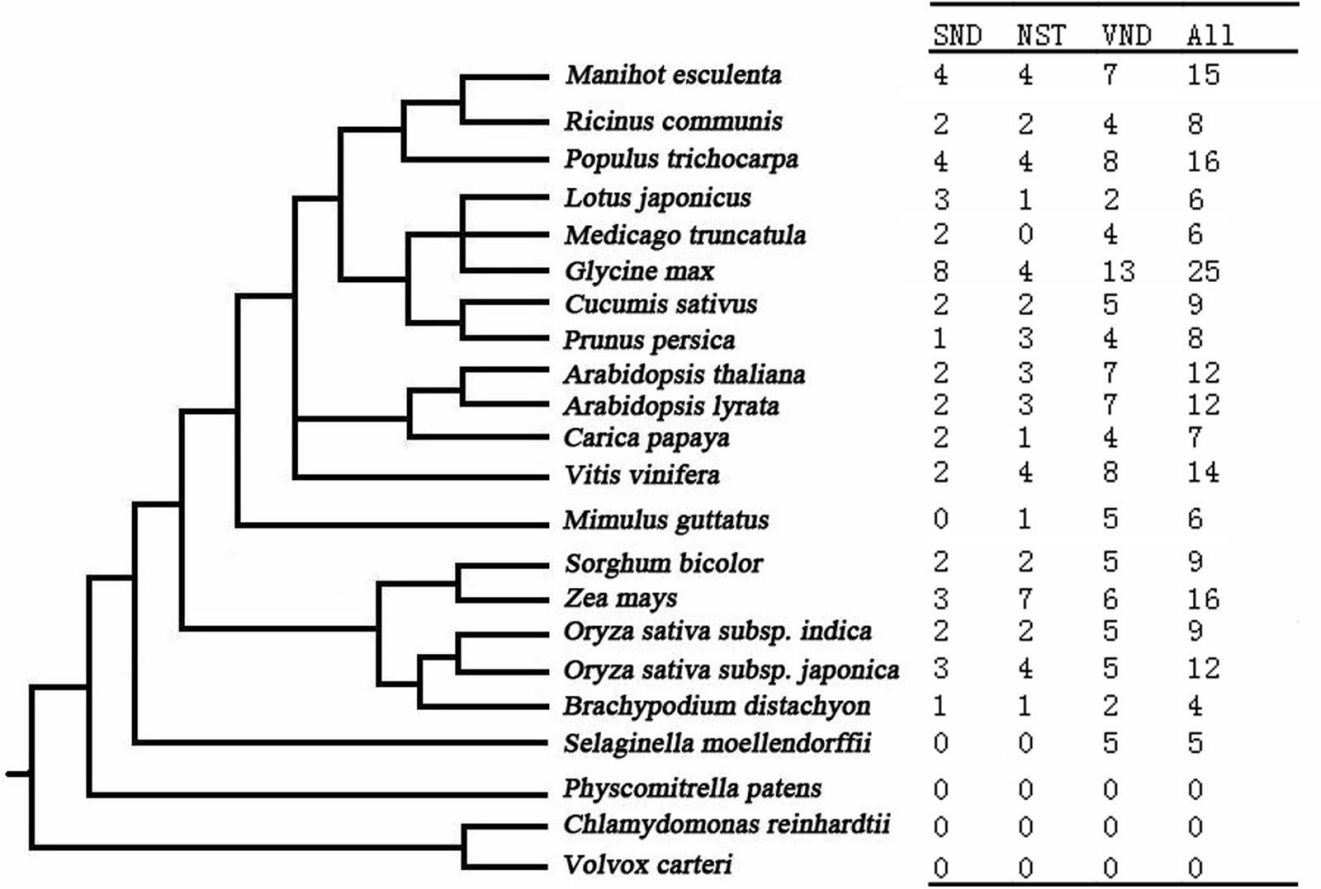
**Phylogenetic tree of 19 different species and summary of number of SND, NST and VND proteins controlling the cell wall composition across these species**.

Plant NAC domain proteins are one of the largest group of plant-specific transcription factors. This study mainly focuses on SND, NST and VNDs. Phylogeny analysis was used to identify the SND, NST and VND members in different species. The NAM domains of all NAC proteins were extracted from the Plant Transcriptional Factor Database http://planttfdb.cbi.pku.edu.cn. Multiple alignments and phylogenetic tree analysis showed that the genes shared the same clades with *Arabidopsis *SND, NST and VND proteins and thus were used for further analysis. This yielded a total of 199 NAC proteins across 19 species that possibly regulate secondary cell wall biosynthesis. The numbers of potential SND, NST and VND proteins with the evolutionary relationship of different species are summarized in Figure 1, while the number of genes in different species may be limited by the Plant Transcriptional Factor Database or sequencing information. For example, MtNST1 was not included in the Plant Transcriptional Factor Database and so was omitted in this study.

The NAC gene family associated with the secondary cell wall biosynthesis evolutionarily first appeared in *S. moellendorffii *(Figure 1). Among the three groups, only VND proteins appeared in *S. moellendorffii*, which was evolutionarily earlier than the other two groups.

### Evolutionary relatedness of SND, NST, VND genes in different species

**Figure 2:**
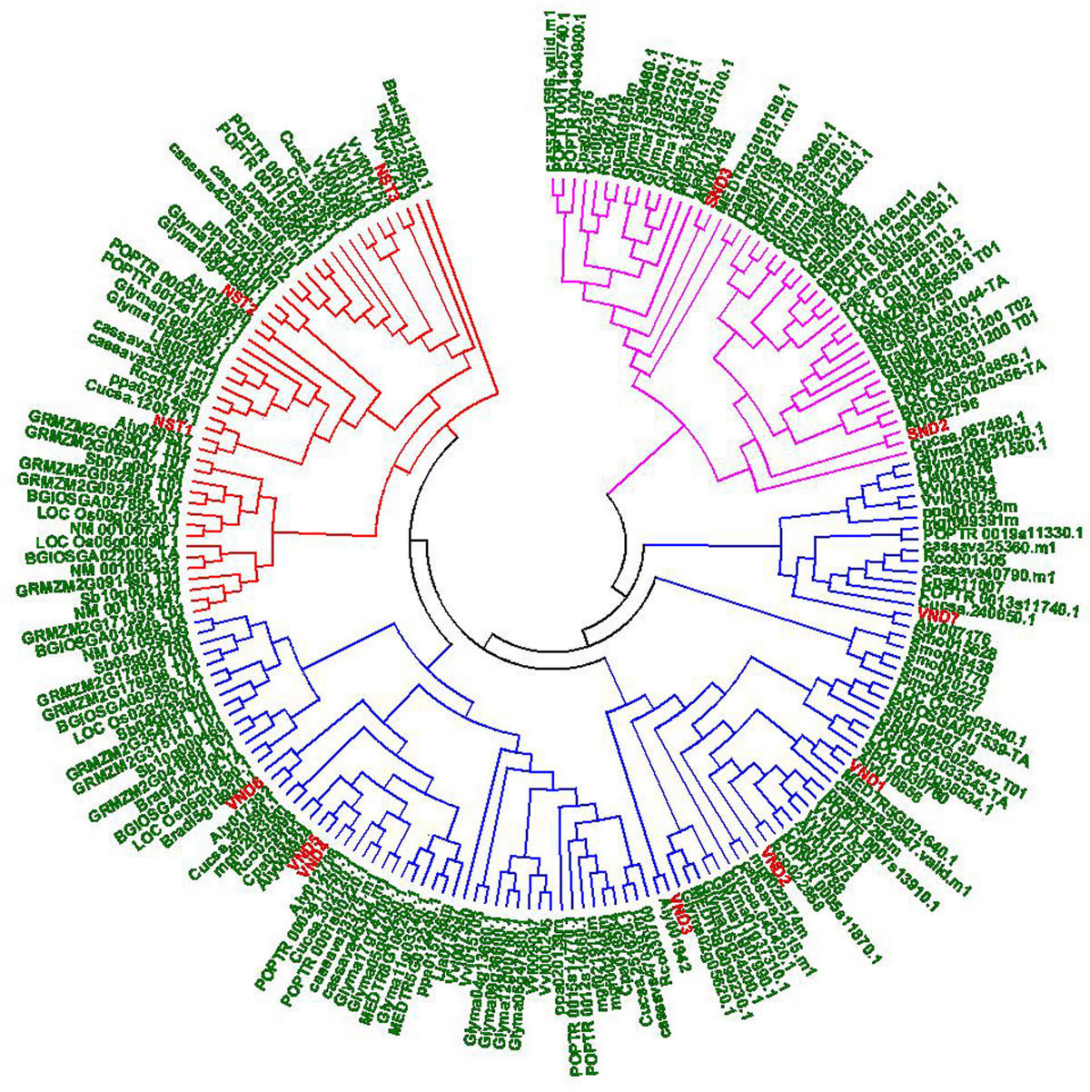
**Phylogenetic tree of SND, NST and VND proteins across the 19 different species**. The three clades are indicated in specific colours. Members of SND, NST, and VND protein from *Arabidopsis *are denoted in red. The detailed information of these proteins was listed in Supplemental Table 3 in additional file 3.

A phylogenetic analysis of the 199 NAC proteins was performed with ClustalX (1.83) using multiple alignment results. A radial NJ tree was generated (Figure 2), and the phylogeny tree was divided into three classes differentiated by specific conserved domains and highlighted with three different colours. The three subgroups (described in Figure 2) were named NST, SND and VND. The orthologues of NSTs (NST1, NST2 and NST3) included 48 proteins from 17 species. Through phylogeny analysis, NST3 (also named as SND1) orthologues were evolutionally closed to NST subfamily. The SND gene subfamily encompasses 45 proteins from 17 species. This subfamily can also be divided into two subunits: the SND2 and SND3 homologues. In the SND3 homologue subunit, all genes were from dicots; whereas the SND2 homologues were from both dicots and monocots. Compared to SND2 and SND3, the VND subfamily was much larger and included four subunits. The first subunit included homologues of VND7, which were all from dicots. Remarkably, the second subunit only contained five proteins from *S. moellendorffii*. In the other two subunits, VND orthologues demonstrated an interspersed distribution from different species.

### Expression patterns of SND, NST and VND genes in *Arabidopsis*

**Figure 3:**
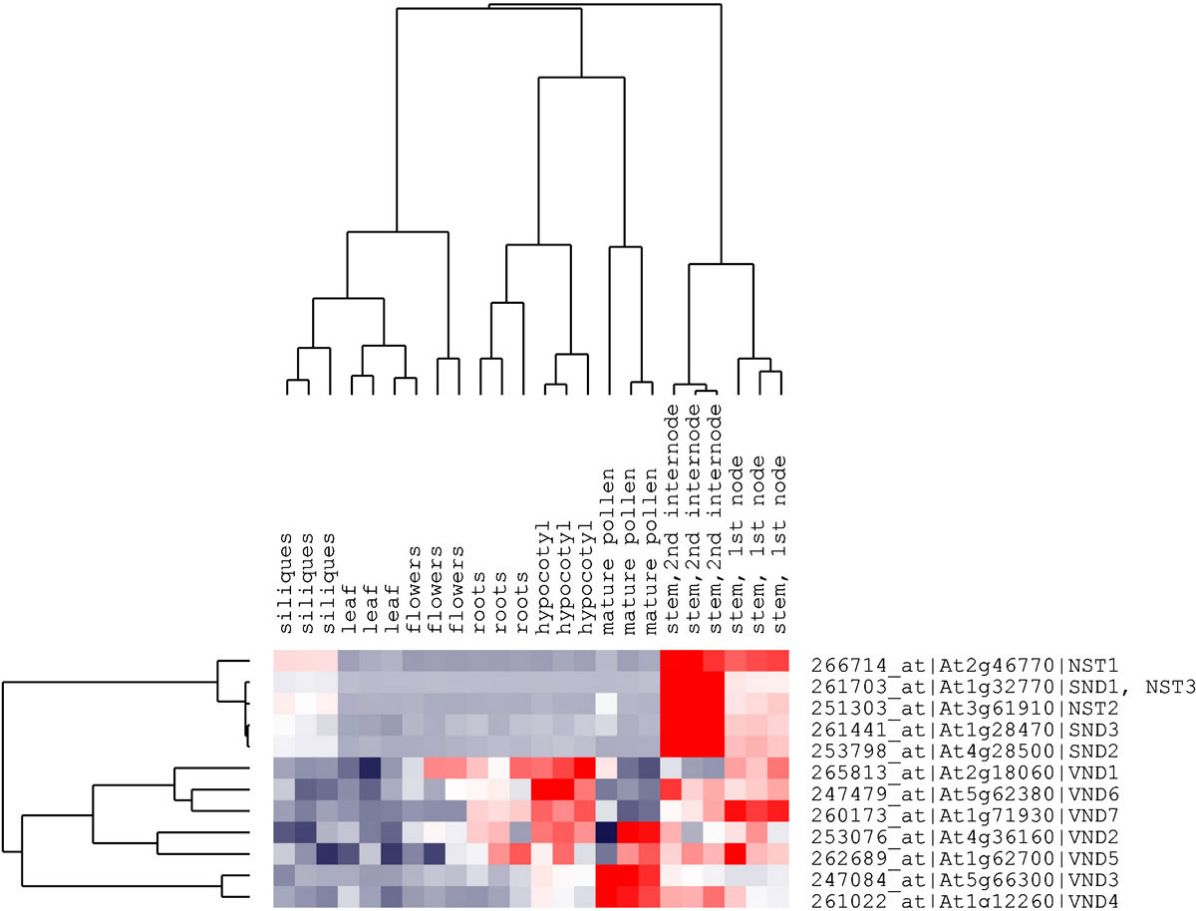
**Cluster analysis of SND, NST and VND genes of *Arabidopsis***. The gene names are indicated on the right; tissue types are indicated above each column. High, average and low levels of expression in a specific tissue are indicated by red, white and blue, respectively.

To better understand the functions of **S**ND, NST and VND genes, we analysed the gene expression profiles of the 12 NAC genes from *Arabidopsis *using microarray data, which included eight diverse *Arabidopsis *tissues with triplicate samples using the *Arabidopsis *ATH1 array [34]. Hierarchical cluster analysis was conducted and genes with similar expression patterns were clustered together. The five NST and SND genes showed a highly similar expression pattern in the tissue specific experiments (Figure 3). They were preferentially expressed in stems, especially the second internodes, and were relatively less expressed in leaves, flowers, roots, hypocotyls and mature pollens. In siliques, these genes were expressed at an average level. In contrast, the VND genes exhibited very different expression profiles in different tissues, except for *VND6 *and *VND7 *which were preferentially expressed in the stem. For example, *VND1 *showed expression in flowers, roots, hypocotyls and first nodes of the stem while *VND3 *and *VND4 *were mainly expressed in mature pollens, and second internodes of the stem. In general, all seven genes were expressed in hypocotyls. With the exception of *VND3 *and *VND4*, the other VND genes were highly expressed in roots. *VND2 *was also highly expressed in mature pollen together with *VND3 *and *VND4*. Although the three classes of NAC genes showed different expression profiles, they all showed lower expression levels in leaves. These microarray results suggest that VND genes may have diverse functions in different developmental stages or cellular processes. The cluster analysis also revealed that several of these transcriptional factors were co-regulated.

### Expression patterns of SND, NST and VND orthologue genes in rice

**Figure 4:**
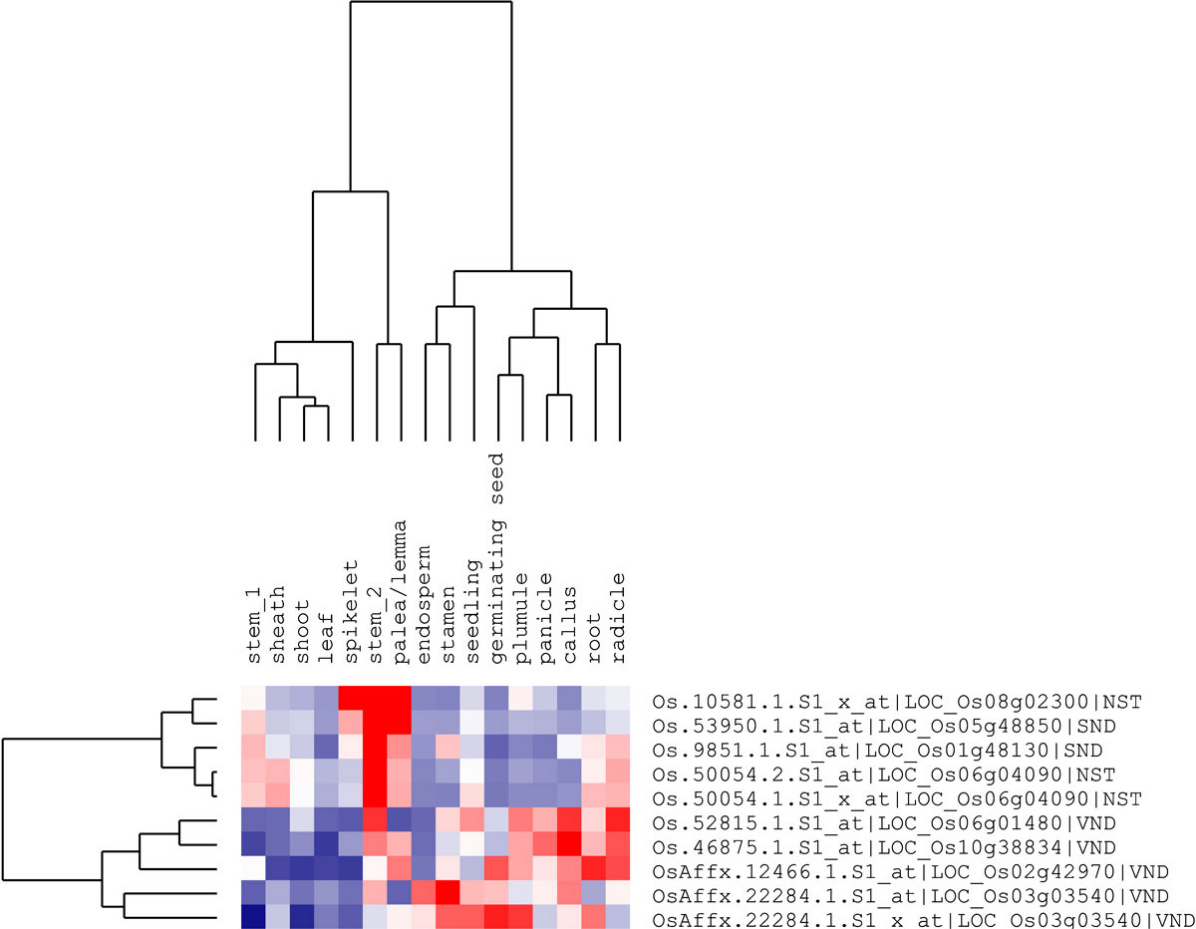
**Cluster analysis of rice SND, NST and VND orthologue genes**. The gene names are indicated on the right; tissue types are indicated above each column. High, average and low levels of expression in a specific tissue are indicated by red, white and blue, respectively.

**Figure 5:**
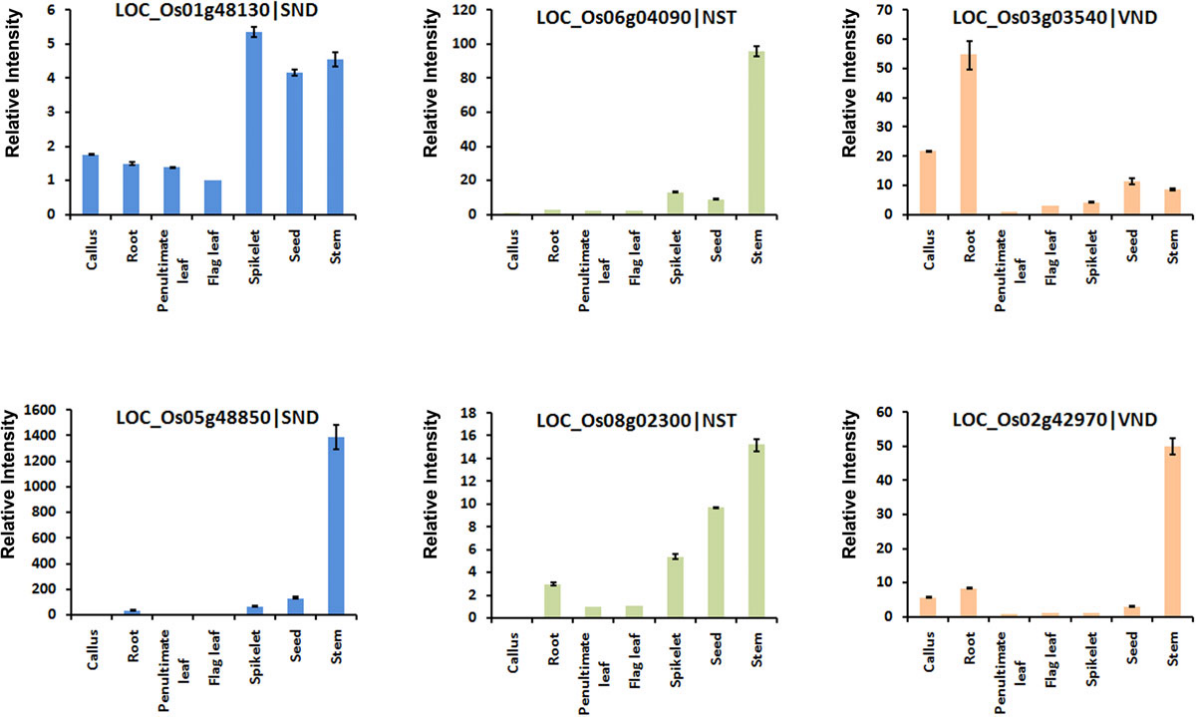
**Real-time RT-PCR analysis of rice SND, NST and VND orthologue genes in different tissues**. NAC gene names are indicated on each chart; tissue names are indicated on the x-axes. The error bars represent the standard deviations of three replicates.

Rice is a model monocot plant for molecular and genetic studies. Recently, large sets of rice tissue-specific microarray data have been available in the GEO database. GSE19024 [35] was selected, including 39 tissues covering the entire tissue culture process and life cycle, from rice *indica *var. Minghui 63, and the gene expression patterns analysed for rice SND, NST and VND orthologue genes that possibly control cell wall composition. Using an approach similar to that employed for *Arabidopsis *microarray analysis, four rice NST and SND orthologue genes showed similar expression pattern in the tissue specific profiles (Figure 4). These four genes were significantly highly expressed in stem tissues, especially in the stem_2 (rice stem during heading stage) and were relatively less expressed in the leaves, endosperm, plumule, panicle and callus. The rice VND orthologue genes exhibited much more diverse expression patterns in these rice tissues. For example, LOC_Os06g04090 showed preferential expression in stem_2, callus and radicle. LOC_Os10g38834 was highly expressed in panicle, callus and radicle. We also analysed the expression profiles of rice SND, NST and VND orthologue genes in the developmental transcriptomes of var. Zhenshan 97, another rice *indica *variety, which showed similar results (data not shown) to those of var. Minghui 63. Real-time RT-PCR experiments were used to further validate the microarray data for those rice NAC genes. Six SND, NST and VND orthologue genes were selected to examine the relative expression intensity in seven rice tissues, including callus, root, penultimate leaf, flag leaf, spikelet, seed and stem. The real-time RT-PCR results (Figure 5) mostly matched the microarray results. The expression pattern analysis of rice SND, NST and VND orthologue genes suggests the existence of conservation of tissue specificity in transcription levels between rice and *Arabidopsis*, especially for SND and NST orthologue genes with preferential expression in developing stem tissues.

### Co-regulatory network analysis for secondary cell wall biosynthesis NAC transcriptional factors SND1, SND2 and SND3

**Figure 6:**
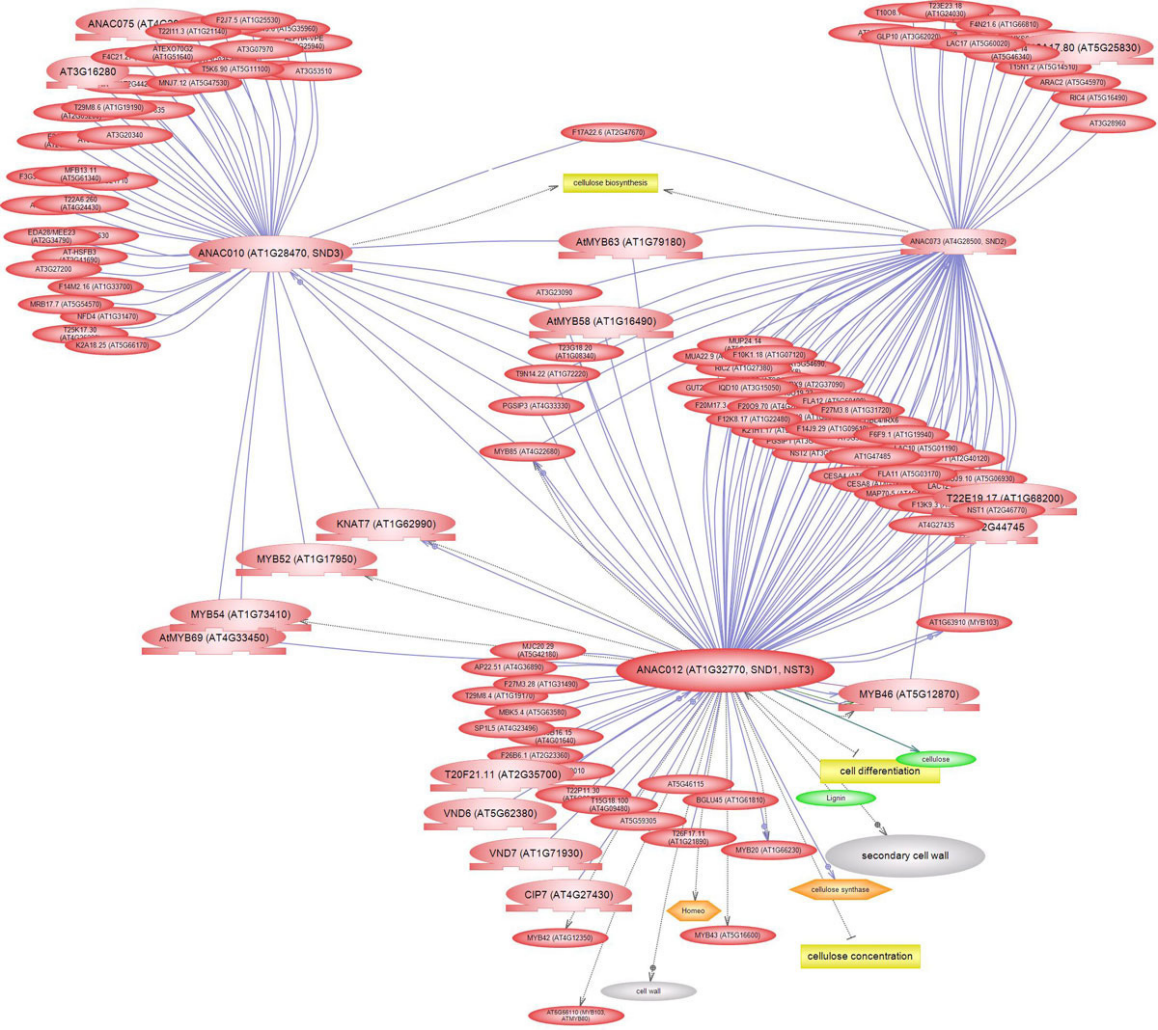
**Co-regulatory network for SND1, SND2 and SND3 in *Arabidopsis***.

The *Arabidopsis *tissue experiment clustering results showed that the SND genes were closely clustered together and displayed a specific expression in second internodes of the stem. The co-expression of genes indicates that they may be co-regulated and involved in similar molecular regulatory pathways. We applied ATTED-II http://atted.jp/ and text mining results to predict the possible network where these three NAC transcriptional factors are involved in secondary cell wall biosynthesis. ATTED-II was built based on 1388 GeneChip data in *Arabidopsis*. The conditions were very diverse, including multiple tissue types, abiotic and biotic stress, hormone treatments and gene mutants [36]. Using Pathway Studio, we built a co-expression gene network for the three SND genes. Each SND gene was co-expressed with multiple other genes (Figure 6). The three SND genes co-expressed with some transcription factor genes, especially the MYB genes, e.g. *MYB46*, *MYB52*, *MYB54*, *MYB58*, *MYB63*, *MYB69*, *MYB85 *and *MYB103*. These MYB transcriptional factors are important in modulating cell wall biosynthesis [22]. For example, *MYB46 *is a direct target of SND1 and a key player in the transcriptional network involved in the regulation of secondary wall biosynthesis in *Arabidopsis *[37]. MYB58 was reported to directly activate the expression of lignin biosynthetic genes and a secondary wall-associated laccase (*LAC4*) gene. MYB58 and MYB63 are transcriptional activators of the lignin biosynthetic pathway during secondary cell wall formation in *Arabidopsis *and their expression was regulated by SND1[38]. The Class II KNOX gene *KNAT7 *was also co-expressed with SND genes. *KNAT7 *was shown to negatively regulate secondary wall formation in *Arabidopsis *and is functionally conserved in *Populus *[39]. Some other transcription factors were also involved in this network, e.g. zinc finger, *ERF38 *and WRKY.

Furthermore, some secondary cell wall metabolism-related genes were co-expressed with SND genes, such as LAC genes (*LAC2*, *LAC5*, *LAC10*, *LAC12 *and *LAC17*), IRX (IRREGULAR XYLEM) genes (*IRX1*, *IRX3*, *IRX6*, *IRX9*, *IRX12 *and *IRX14*), *CESA4 *(CELLULOSE SYNTHASE A4) and pectinase related protein. The knockout mutant of *LAC2 *had been reported to reduce root elongation under PEG-induced dehydration [40] and *LAC17 *mutants appear to have a reduced deposition of G lignin units [41]. *IRX1 *encodes a member of the cellulose synthase family [42-44], *IRX3 *encodes a xylem-specific cellulose synthase [45], *IRX6 *encodes a member of the COBRA family (similar to phytochelatin synthetase) [46], *IRX9 *encodes a putative family 43 glycosyl transferase [47,48], *IRX12 *(also known as *LAC4*) appears to have laccase activity [41], *IRX14 *encodes a putative family 43 glycosyl transferase [48,49], *CESA4 *encodes a cellulose synthase [46,50], and all these genes are involved in secondary cell wall biosynthesis.

Interestingly, several RIC (ROP-INTERACTIVE CRIB MOTIF-CONTAINING PROTEIN) genes were also co-regulated with SND genes, e.g. *RIC2 *(involved in pollen tube growth and function [51]) and *RIC4 *(interacts with ROP2 during pavement cell morphogenesis and with ROP1 to promote apical F-actin assembly [52]). In addition, the co-regulatory network analysis also revealed that many novel genes were co-expressed with SNDs.

There were a total of 134 genes involved in this network (Supplemental Table 2 in additional file 2). GO analysis [30] was also performed for these 134 SND co-regulated genes (Table 1) to decipher the possible biological pathways in which these genes were involved. Of the 134 genes queried, there were 131 genes with annotated GO items. We used 0.05 as the cut-off of FDR adjusted p-value. The most significantly enriched GO terms were 'secondary cell wall biogenesis process' (GO:0009834, FDR p-value = 2.40E-22), 'cellular polysaccharide biosynthetic process' (GO:0033692, FDR p-value = 4.30E-07), 'phenylpropanoid biosynthetic process' (GO:0009699, FDR p-value = 2.50E-06), 'transcription factor activity' (GO:0003700, FDR p-value = 3.00E-49) and 'laccase activity' (GO:0008471, FDR p-value = 3.00E-09). The GO terms related to other biological processes were also enriched, e.g. 'response to stress' (GO:0006950, FDR p-value = 5.60E-03), 'oxidoreductase activity' (GO:0016491, FDR p-value = 4.00E-04), 'transition metal ion binding' (GO:0046914, FDR p-value = 3.50E-05) and transporter activity. Also, most genes were localized in nuclear and membrane parts.

Co-regulatory network analysis of SNDs and GO enrichment analysis indicated that most co-expressed genes were involved in secondary cell wall biogenesis, while we also found that some oxidoreductase activity and phenylpropanoid biosynthesis pathway genes were co-expressed with SND genes, e.g. peroxidase 64, NADPH oxidase and *FLS2*. Some processes such as carbohydrate synthesis and transport were coordinately regulated toward cell wall biosynthesis. There may be cross-talk between secondary wall biosynthesis and other biological processes.

## Conclusions

Combining the bioinformatics data mining and bench validation approach, we analysed the NST, VND and SND genes across plant species. The comparative genomic analysis revealed that the group VND of the NAC gene family evolutionarily first appeared in *S. moellendorffii*. The *Arabidopsis *and rice gene expression analysis showed the specific patterns of these NAC genes and the conservation of SNDs and NSTs in *Arabidopsis *and rice, and they were preferentially expressed in stems. The gene network analysis of SND genes in *Arabidopsis *showed that three SND genes (*SND1*, *SND2 *and *SND3*) co-expressed with multiple transcription factor genes, especially MYB genes and *KNAT7*, which are important in modulating cell wall biosynthesis. Additionally, the co-regulatory network analysis revealed many novel genes and pathways that could potentially be involved in cell wall biosynthesis and regulation. Nevertheless, there may be cross-talk between secondary wall biosynthesis and other biological process, such as stress response.

In summary, these results provided new insight into the evolution and the gene regulatory network of a subgroup of the NAC gene family controlling cell wall composition from the perspective of bioinformatics. These may help us to better understand the possible molecular mechanism underlying the regulation network of secondary cell wall biosynthesis and, therefore, improve plant biomass production.

## Competing interests

The authors declare that they have no competing interests.

## Authors' contributions

WX, ZS and JY conceived and designed the study. QW performed the experiments. WX and DY analysed the data. WX, DY, ZS and RDS wrote the paper.

## Supplementary Material

Additional file 1 **Supplemental Table 1**. Primer list of rice SND, NST and VND orthologue genes for real-time RT-PCR (in doc format). Click here for file

Additional file 2 **Supplemental Table 2**. The annotation of 134 genes involved in *Arabidopsis *SNDs-involved network (in xls format). Click here for file

Additional file 3 **Supplemental Table 3**. The detailed information of 199 SND, NST and VND proteins studied across the 19 different species in this work (in xls format). Click here for file
