# Supplementary material for: Comparative genomic analysis of NAC transcriptional factors to dissect the regulatory mechanisms for cell wall biosynthesis
Source: BMC Bioinformatics. 2012 Sep 11;13(Suppl 15):S10. doi: 10.1186/1471-2105-13-S15-S10 (PMC3439729; doi:10.1186/1471-2105-13-S15-S10)
Supplement: Additional file 1 — Supplemental Table 1. Primer list of rice SND, NST and VND orthologue genes for real-time RT-PCR (in doc format). [file 1471-2105-13-S15-S10-S1.doc]

**Supplemental Table 1. Primer list of rice NAC genes for real-time RT-PCR**

| **Locus ID** | **Forward** | **Reverse** |
| --- | --- | --- |
| LOC_Os01g48130 | TTCTGCTCATTTGACGATGC | TTTTCTTCCATCCATCAGGG |
| LOC_Os05g48850 | ACTACTCCATCGTCCATGGC | GAACTCGTGCACAACGTTTG |
| LOC_Os06g04090 | TGACGATGGAGATGATGCAT | CATGCAATATGCGTAGACCG |
| LOC_Os08g02300 | CGTGGCACTGTAACACATGA | ACTAGCAGCTAGGGTGGCAG |
| LOC_Os03g03540 | TTCTCAACAGCGACGAGAGA | CAAACACACAGATTCCGAGG |
| LOC_Os02g42970 | GTCCATGTCCAGCTACGGAT | GTCGAAGCGTAGTCCAAAGC |
